# In-line filtration of intravenous infusion may reduce organ dysfunction of adult critical patients

**DOI:** 10.1186/s13054-019-2618-z

**Published:** 2019-11-22

**Authors:** Elke Schmitt, Patrick Meybohm, Eva Herrmann, Karin Ammersbach, Raphaela Endres, Simone Lindau, Philipp Helmer, Kai Zacharowski, Holger Neb

**Affiliations:** 1 Department of Anaesthesiology, Intensive Care Medicine and Pain Therapy, University Hospital Frankfurt, Frankfurt am Main, Germany; 2 Institute of Biostatistics and Mathematical Modelling, Department of Medicine, Goethe University Frankfurt, Frankfurt am Main, Germany; 3 Department of Anaesthesiology, University Hospital Wuerzburg, Wuerzburg, Germany; 4 Division of Software and Information Systems, Department of Information and Communication Technology, University Hospital Frankfurt, Frankfurt am Main, Germany

**Keywords:** Intensive care, Infusion management, In-line filtration, Particles, Organ dysfunction, Inflammation

## Abstract

**Background:**

The potential harmful effects of particle-contaminated infusions for critically ill adult patients are yet unclear. So far, only significant improved outcome in critically ill children and new-borns was demonstrated when using in-line filters, but for adult patients, evidence is still missing.

**Methods:**

This single-centre, retrospective controlled cohort study assessed the effect of in-line filtration of intravenous fluids with finer 0.2 or 1.2 μm vs 5.0 μm filters in critically ill adult patients. From a total of *n* = 3215 adult patients, *n* = 3012 patients were selected by propensity score matching (adjusting for sex, age, and surgery group) and assigned to either a fine filter cohort (with 0.2/1.2 μm filters, *n* = 1506, time period from February 2013 to January 2014) or a control filter cohort (with 5.0 μm filters, *n* = 1506, time period from April 2014 to March 2015). The cohorts were compared regarding the occurrence of severe vasoplegia, organ dysfunctions (lung, kidney, and brain), inflammation, in-hospital complications (myocardial infarction, ischemic stroke, pneumonia, and sepsis), in-hospital mortality, and length of ICU and hospital stay.

**Results:**

Comparing fine filter vs control filter cohort, respiratory dysfunction (Horowitz index 206 (119–290) vs 191 (104.75–280); *P* = 0.04), pneumonia (11.4% vs 14.4%; *P* = 0.02), sepsis (9.6% vs 12.2%; *P* = 0.03), interleukin-6 (471.5 (258.8–1062.8) ng/l vs 540.5 (284.5–1147.5) ng/l; *P* = 0.01), and length of ICU (1.2 (0.6–4.9) vs 1.7 (0.8–6.9) days; *P* <  0.01) and hospital stay (14.0 (9.2–22.2) vs 14.8 (10.0–26.8) days; *P* = 0.01) were reduced. Rate of severe vasoplegia (21.0% vs 19.6%; *P* > 0.20) and acute kidney injury (11.8% vs 13.7%; *P* = 0.11) was not significantly different between the cohorts.

**Conclusions:**

In-line filtration with finer 0.2 and 1.2 μm filters may be associated with less organ dysfunction and less inflammation in critically ill adult patients.

**Trial registration:**

The study was registered at ClinicalTrials.gov (number: NCT02281604).

## Background

The intravenous administration of both fluids and drugs represents one of the main therapy pillars within intensive care medicine. Contamination with particles, however, and their potential harmful effects, especially for critically ill patients is a suspected issue [1–4]. Recent studies [5–7] showed that the particle load is significantly higher without in-line filters and that in-line filtration may be an effective tool in preventing particle contamination to patients. Today, at least three different commercial in-line filter systems are available. The 5.0-μm in-line filter reduces rough particles (glass, rubber, plastic). The potential advantage of the finer positively charged 0.2 and 1.2 μm in-line filters is that they can hold back not only glass, rubber, and plastic particles but also particles from drug incompatibilities, air, microorganisms (bacteria size 1–3 μm), and smaller endotoxins [8]. Therefore, catheter-related bloodstream infections are a potential target of prevention via in-line filtration, too.

In general, potential systemic effects of microparticles on different organs may depend on material, size, amount, and patient population. Recent studies focussed on critically ill children and new-borns showing a significant reduction of complications, e.g. thrombosis, sepsis, and organ failure as well as a reduction of length of stay in ICU [9–12], while others [13, 14] found no benefits. Furthermore, post-mortem studies on patients with acute respiratory distress syndrome (ARDS) suggest a harmful effect of those particles especially for the lungs and have shown that infusion therapy can lead to a particle-induced mechanic vascular occlusion and to intravascular formation of foreign bodies [15–18]. In vitro studies demonstrated a particle-induced modulation of the immune system [19]. The German Society of Anaesthesiology and Intensive Care Medicine and the Berufsverband Deutscher Anästhesisten both strongly suggest the use of particle filters with size always adapted to the type of fluid or drug (chosen as small as possible due to the suspected harmful effects of very small particles in the range of 2–100 μm) [20]. Organisations such as the PDA (Parenteral Drug Association) Europe offer regular training for physicians to transfer actual knowledge about potential harmful effects of particles for patients and also participate actively in research activities on particles in parenteral drugs. But the evidence on the benefits for adult intensive care patients is still unclear.

The aim of this study was to elucidate if the use of the finer 0.2 or 1.2 μm in-line filters was superior in comparison to the larger 5 μm in-line filter within the framework of i.v. fluid and drug management for adult critical ill patients.

## Materials and methods

### Study design

This single-centre, retrospective controlled cohort study covering the period between February 2013 and April 2015 assessed the effects of in-line filtration of intravenous fluids on the reduction of complications in critically ill adult patients of a tertiary German hospital.

Approval of the study protocol was obtained from the local ethics committee (Goethe-University Frankfurt am Main, ref. 8/14 from 30 January 2014). The study was registered at ClinicalTrials.gov (NCT02281604). Funding was exclusively provided by internal departmental funding.

### Patient enrolment and cohorts

All adult (age ≥ 18 years) critical patients who were admitted to the University Hospital Frankfurt between February 2013 and March 2015 and had at least 1 admission to the 34-bed intensive care unit (ICU) were included. Admission to the ICU for more than one time during the same hospital stay was not an a priori exclusion criteria, and the information of these ICU stays was cumulated. Patients admitted to the hospital more than one time during this period (ranging from returns after weeks to months or years) were not excluded from the study, but only the first hospital stay was used.

**Fig. 1:**
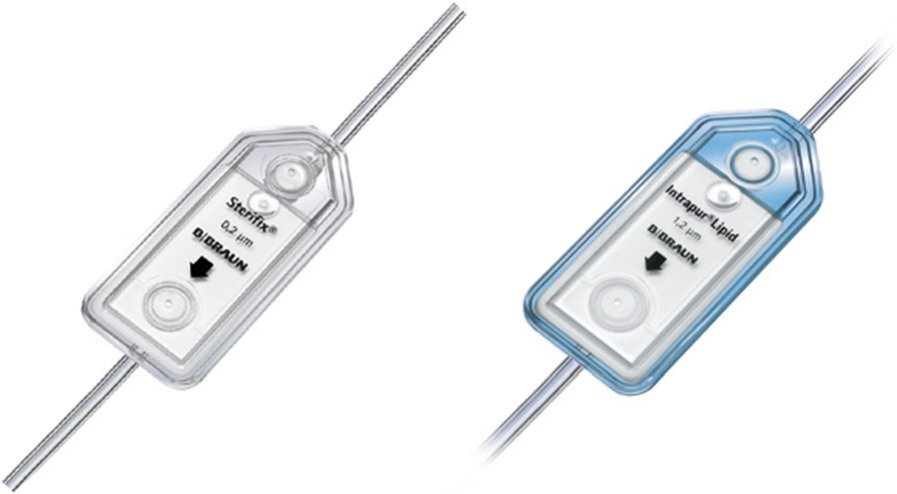
Fine in-line filters 0.2 μm Sterifix for aqueous solutions (left) and 1.2 μm Intrapur for lipid-containing mixtures (right) (from www.bbraun.de)

**Fig. 2:**
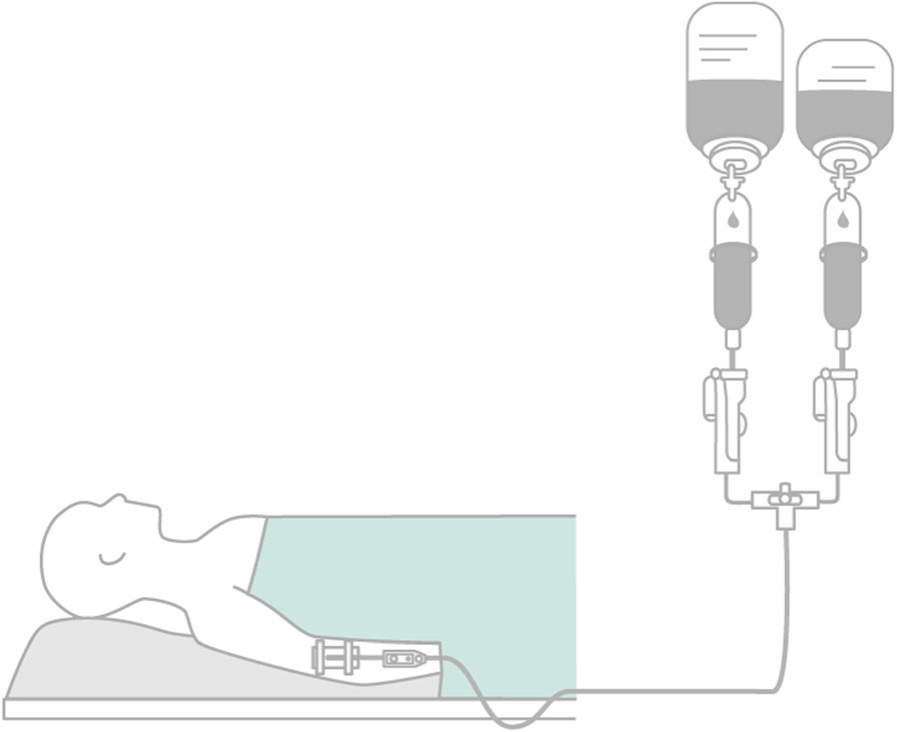
Scheme of in-line filter inserted into the IV lines (from www.bbraunforsafety.com)

Until February 2013, no in-line filters were used routinely on the ICU, intraoperatively or on the regular wards. To reduce the risk of particle contamination, larger 5 μm in-line filters have been routinely introduced intraoperatively and on all normal care stations from February 2013 on up to the present. On the ICU instead, in the period from February 2013 to January 2014, finer 0.2 or 1.2 μm in-line filters were routinely used (0.2 μm Sterifix for aqueous solutions and 1.2 μm Intrapur for lipid-containing mixtures) (Fig. 1). In February 2014, filter systems on the ICU were again switched to the larger 5 μm in-line filters during a cost-reduction initiative (about 15 € per 0.2 or 1.2 μm in-line filter and doubts regarding the efficacy of the finer filters), and the remaining 0.2 or 1.2 μm in-line filters were consumed in parallel. Thus, since April 2014, only larger 5 μm in-line filters have been used on the ICU. The peripheral lines were protected equally as the central lines by the respective in-line-filters of each time period and ward. Usually, the filters were placed directly behind the three-way cock of the (central) venous line (Fig. 2).

While patients admitted to the ICU between February 2013 and January 2014 were assigned to the fine filter cohort (finer 0.2 or 1.2 μm in-line filters; *n* = 1621), patients admitted between April 2014 and March 2015 were assigned to the control filter cohort (larger 5 μm in-line filters; *n* = 1594).

To account for differences in patient characteristics (exact matching for surgery group and sex, nearest neighbour matching with respect to age), a propensity score matching was performed, which resulted in the final number of *n* = 1506 subjects for both cohorts.

No dropout occurred as routine data could be followed up until hospital discharge and no patient consent was needed.

### Endpoints

Microparticles are suspected to be responsible for inflammation of any kind that could lead to different organ manifestations. Inflammation on its own can cause vasodilatation via increased NO production, possibly leading to severe vasoplegia, also known as vasodilatory shock. As this complex syndrome can in fact only be assessed clinically by the vasopressor dosage necessary to maintain an acceptable mean arterial blood pressure, we tried to identify patients suffering from this syndrome by choosing high dose norepinephrine as one of the most appropriate potential surrogate parameters [21].

Primary endpoint of this epidemiological study was the rate of severe vasoplegia [22–24] defined by a continuous norepinephrine rate ≥ 0.3 μg/kg/min for at least 1 min. Patients that did not receive any administration of norepinephrine reported in the data files were counted as patients with dose zero due to the fact that only records exist if patients did receive norepinephrine. Values of norepinephrine rates > 1.0 μg/kg/min were discarded as unrealistic on our ICU. In addition, we analysed the rate of patients receiving norepinephrine and also methylene blue (which is an additional option to treat vasoplegia) to obtain a more comprehensive overview on this endpoint.

The following secondary endpoints were recorded until hospital discharge:

(i) Multiple-organ dysfunction (assessed by maximal SOFA score, additionally SAPS II and TISS-10/TISS-28).
(ii) Incidence and severity of lung dysfunction (defined by minimal Horowitz index (obtained by the ratio of the values of paO_2_ and FIO_2_) and ARDS score [25] (obtained by calculating the scores with the standard definition from the minimal Horowitz index of each patient (0–100 = severe ARDS (score 3), 101–200 = moderate ARDS (score 2), 201–300 = mild ARDS (score 1), 301–… = no ARDS (score 0))). Information on duration of mechanical ventilation or airway pressures was not present.
(iii) Incidence and severity of acute kidney injury (AKI) was defined by a modified AKIN [26] score based only on creatinine values before the first dialysis without information on urine output (0 = no AKI, 1 = mild AKI, 2 = moderate AKI, 3 = severe AKI) and by the dialysis rate as albuminuria and glomerular filtration rate were not available in this retrospective study to analyse the current recommended score from KDIGO (Kidney Disease Improving Global Outcome) (https://kdigo.org/).
(iv) Incidence of delirium (maximal delirium score defined by ICDSC standard screening).
(v) Grade of inflammation (by maximal interleukin-6 (IL-6) values [27, 28]). Interleukin-6 levels were dosed routinely every 6 or at least 12 h (at 4 am and 4 pm) in our intensive care unit and, according to specific individual needs for a critical patient, additionally also occasionally at individual extra time points in between, whereas other inflammation markers, e.g. C-reactive protein or procalcitonin, were not determined routinely. Blood samples were sent to the central lab where an electro-chemiluminescence immunoassay (ECLIA) test was performed. The measurement usually takes 30–60 min in case of an emergency blood sample. As any unspecific activation of macrophages generally leads to an elevation of IL-6 levels in patients’ serum at first, this marker is sensitive in detecting ongoing inflammation [29].
(vi) Length of stay on ICU (cumulated time in days defined by admission/discharge dates and times) and in hospital (time in days defined by admission/discharge date and time).
(vii) In-hospital mortality over 28 days and over total hospital stay (defined by discharge code).
(viii) Rate of in-hospital complications and morbidities (acute myocardial infarction, acute ischemic stroke, pneumonia, sepsis, defined by their ICD-10 codes from diagnoses).
(ix) Incidence and severity of lung/cardiac dysfunction (assessed by rate of ECMO and cumulated ECMO time, defined by start/end dates and times).

Patients who need higher norepinephrine rates might also suffer from sepsis or septic shock, but especially, inflammation after cardiopulmonary bypass triggers vasoplegia as well. In contrast, the diagnosis sepsis is reasonably different from inflammation and vasoplegia; therefore, we analysed these two endpoints independently.

### Data collection and statistical analysis

Routine data were obtained from the hospital information systems ORBIS (AGFA HealthCare, Bonn, Germany) [30] and the electronic patient data management system Metavision (iMDsoft, Tel Aviv, Israel) [31], which was first established in 2013 on the ICU. No additional study documentation on CRFs/eCRFs was performed. The documentation of the patient-related medical data was performed by using a pseudonymised patient ID or case number. No characteristics that would allow the direct identification of certain patients during the data analysis were transmitted. The study data were protected from foreign access, and only members of the study team had access to it.

The exported data were screened for apparent inconsistencies (e.g. inverted start/end dates, values being clearly out of the normal reference range by many orders) and missing information (e.g. type of discharge, measurements, medication) to detect documentation errors. Missing information was corrected manually, where possible, based on the original full patient file from the source hospital information systems AGFA ORBIS or Metavision. Patients with missing laboratory or score values for an endpoint were only excluded from that endpoint but not from the study. For patients not mentioned in the therapeutic intervention data (e.g. norepinephrine, dialysis), it was assumed that those patients had no intervention (e.g. zero doses, no dialysis). For patients with cardiac surgery, the potential event of myocardial infarction was not taken into account, as well as for patients with neurosurgery not the potential event of ischemic stroke (such patients were treated as having no event) to exclude potential pre-existing diseases.

The information about different ICU stays during the same hospital stay was then summarised, and patients were assigned to either the fine filter cohort (if all ICU stays were during the fine filter period), the control cohort (if all ICU stays were during the control filter period), or excluded (if neither of the two).

Statistical analysis was performed with the use of the statistic software R (R Foundation for Statistical Computing, Vienna, Austria) version 3.4.1 [32] with the package “MatchIt” version 3.0.2 for propensity score matching. Additional analysis was performed with BiAS (epsilon-Verlag, Frankfurt/Main, Germany, maintained by Institute of Biostatistics, Goethe University Frankfurt, Germany) for Windows version 11.06 [33]. Although if due to the large number of patients per cohort that depended only on the time period, the patient characteristics were most likely already approximately equally distributed in each cohort, we aimed to minimise potential bias and effects in the outcomes as best as possible. Therefore, in the total cohort of *n* = 3215 patients, a propensity score matching adjusting pairwise for the main characteristics—surgery group, sex, and age in each cohort—was introduced that lead to a total of *n* = 3012 patients (*n* = 1506 each per fine filter and control filter cohort). The propensity score matching was performed completely independent from the later outcome variables (such as Horowitz, creatinine, IL-6), and especially, no patient selection was done. For other minor characteristics (such as scores or baseline values), no matching was performed, as this would need an even much higher amount of patients to match for more characteristics equally in both cohorts. Further pre-tests (Shapiro-Wilk) were performed to determine if any of the endpoints or their logarithmised values followed a normal distribution. As deviations from normal distribution could be confirmed in all tests for the continuous endpoints, the Mann-Whitney *U* tests were performed for group comparisons.

There was no need to perform any significance correction for multiple testing, as our scope was to analyse the outcomes for different secondary endpoints independently from each other and from the main endpoint.

The main hypothesis was to test if the use of the finer 0.2 or 1.2 μm in-line filters was superior in comparison to the larger 5 μm in-line filter with respect to the incidence rate of severe vasoplegia.

For sample size estimation, a risk reduction of 20% by the usage of the finer 0.2 or 1.2 μm in-line filters from an incidence rate of 10 to 8% for the primary endpoint was considered as clinically relevant. This difference could be detected with a chi-square test with at least 80% power and a significance level of α ≤ 0.05, if about *n* = 1400 subjects were enrolled in each cohort, as calculated by G*Power (Heinrich Heine University of Duesseldorf, Germany) [34] in the original study analysis plan. The evaluation of the therapy effect was performed by a logistic regression (with regressor variables age, sex, surgery group, and cohort), and the test decision (for difference between the cohorts) was carried out with the Wald test for the coefficients of the therapy effect with R (package “survey” version 3.33). The risk reduction was quantified as odds ratio (OR) with 95% confidence interval.

The secondary hypotheses were to test if the use of the finer 0.2 or 1.2 μm in-line filters was superior to the use of the larger 5 μm in-line filter regarding the incidence of the secondary endpoints. Risk reduction was quantified as risk ratio (RR) with 95% confidence interval. Both cohorts were compared regarding the differences of incidence rates and their 95% confidence interval (CI), of mean ± standard error (SE) for continuous or median values with quartiles for at least ordinal endpoints. Differing from that, median values were also used for continuous endpoints if their distribution was not approximately symmetric.

In addition, appropriate tests (exact Fisher or chi-square for rates and Mann-Whitney *U* for all other endpoints) for significance were performed. Effect size was reported as risk ratio (range from 0 to infinity) for binary endpoints and by the Mann-Whitney *U* (MW) estimator (range from 0 to 1) for at least ordinal endpoints, each with corresponding 95% CI. Where appropriate, additionally, also linear or logistic regression models dependent on cohort, age, sex, surgery group, and baseline values were used to perform a Wald test for significance of the cohort and the related odds ratio was reported where appropriate. The latter models provide the advantage of considering potential influence factors (e.g. baseline values) but are less robust than non-parametric tests, on which we primarily based our results.

## Results

Forty-one percent of the matched patients underwent only cardiac surgery (and additionally about 22% of the total surgeries in the mixed surgery group were cardiac surgeries, with about 53% of the patients in the mixed group undergoing cardiac surgery in addition to other surgery). The matched cohorts with *n* = 1506 patients each showed no significant differences in the baseline characteristics except a higher age, SAPS II score, and delirium score in the finer filter cohort (Table 1)

**Table 1:** Baseline characteristics of patients (propensity score matched)

	Fine filter cohort (*n* = 1506)	Control filter cohort (*n* = 1506)	*P* value^a^
Age (years)	68 (58.0–75), 65.7 ± 0.3	66 (57–74), 63.9 ± 0.3	Approximate match, *P* < 0.01, MW 0.54 (0.52–0.56)
Male sex (*n*; %)	1081; 71.8%	1081; 71.8%	Exact match, *P* = 1.00
Surgery groups (*n*; %)			Exact match, *P* = 1.00
No surgery	204; 13.5%	204; 13.5%	
Dermatology, ophthalmology	4; 0.3%	4; 0.3%	
Neurosurgery	1; 0.1%	1; 0.1%	
Otorhinolaryngology	16; 1.1%	16; 1.1%	
Thoracic	14; 0.9%	14; 0.9%	
Cardiac	617; 41.0%	617; 41.0%	
Vascular	48; 3.2%	48; 3.2%	
Visceral and endocrine	46; 3.1%	46; 3.1%	
Urology	12; 0.8%	12; 0.8%	
Gynaecology	1; 0.1%	1; 0.1%	
Obstetric	3; 0.2%	3; 0.2%	
Oral and maxillofacial	2; 0.1%	2; 0.1%	
Trauma/orthopaedic	32; 2.1%	32; 2.1%	
Other surgery	1; 0.1%	1; 0.1%	
Mixed	505; 33.5%	505; 33.5%	
Discarded for matching (n)	115	88	
Initial SOFA score^b^	8 (6–11)	8 (5–11)	> 0.20
Initial SAPS II^c^	46 (33.5–58)	41 (30–56)	< 0.01, MW 0.56 (0.52–0.59)
Initial TISS-10^d^	18 (14–26)	21 (14–26)	> 0.20
Initial TISS-28^e^	39 (33–45)	38 (32.5–45)	0.17
Initial Horowitz index^f^	317 (234–393)	314 (232–383)	> 0.20
Initial creatinine value (mg/dl)^g^	0.90 (0.72–1.17)	0.90 (0.72–1.20)	> 0.20
Initial delirium score^h^	2 (0–5)	1 (0–3)	< 0.01, MW 0.55 (0.51–0.59)
Initial interleukin-6 value (ng/l)^i^	284.2 (135.9–605.2)	284.2 (131.2–672.0)	> 0.20

This table shows the distribution of subjects between the fine filter and control filter cohort by demographic characteristics, surgery category, and baselines for multi-organ scores and for chosen laboratory values at admission. Age, delirium score, and SAPS II at admission were significantly worse for the fine filter cohort

Data are presented as the mean ± standard error, as the median (first quartile–third quartile), as the percentage rates (with 95% confidence intervals), or as the number (*n*) of patients, where indicated

^a^*P* values were calculated using the Wilcoxon-Mann-Whitney *U* test for equality of means, Pearson’s chi-square test, or Fisher’s exact test, as appropriate. Odds ratios or Mann-Whitney effect estimators (Delong method for AUC between 0 and 1) are provided as appropriate and only if the *P* value is significant

^b^Available patients with SOFA score (%): 30.2 (fine filter) and 39.6 (control filter)

^c^Available patients with SAPS II score (%): 36.6 (fine filter) and 44.4 (control filter)

^d^Available patients with TISS-10 score (%): 32.3 (fine filter) and 43.0 (control filter)

^e^Available patients with TISS-28 score (%): 32.3 (fine filter) and 43.0 (control filter)

^f^Available patients with Horowitz index (%): 86.8 (fine filter) and 72.8 (control filter)

^g^Available patients with creatinine value before dialysis (%): 99.1 (fine filter) and 99.1 (control filter)

^h^Available patients with delirium score (%): 20.5 (fine filter) and 52.5 (control filter)

^i^Available patients with interleukin-6 value (%): 98.4 (fine filter) and 98.2 (control filter)

### Primary endpoint

We found no significant difference in the incidence rate of severe vasoplegia between the fine filter and the control filter cohort (21.0% vs 19.6%; risk ratio (95% CI) = 1.07 (0.93–1.23), *P* > 0.20 for non-parametric Fisher test; *P* > 0.20 for Wald test on regression model with odds ratio (95% CI) = 1.05 (0.87–1.27)) (Table 2), although the rate of patients receiving norepinephrine in the fine filter cohort (82.9% vs 65.4%; *P* <  0.01) and the maximum rate of norepinephrine per patient in the fine filter cohort were significantly higher (median 0.09 vs 0.05; *P* <  0.01) (Table 2). Interestingly, the calculated rate of vasoplegia in both cohorts was here about twice as high as assumed in the SAP. The additionally analysed methylene blue rate was not significantly different between the two cohorts (9.8% vs 10.2%; *P* > 0.20) (Table 2).

**Table 2:** Primary outcome parameters (vasoplegia)

	Fine filter cohort (*n* = 1506)	Control filter cohort (*n* = 1506)	*P* value^a^	*P* value^b^
Vasoplegia (*n*; %)	316; 21.0% (19.0–23.1%)	295; 19.6% (17.6–21.7%)	> 0.20, RR 1.07 (0.93–1.23)	> 0.20, OR 1.05 (0.87–1.27)
Maximum rate of norepinephrine (μg/kg/min)	0.09 (0.03–0.23), 0.16 ± 0.01	0.05 (0.00–0.20), 0.14 ± 0.01	< 0.01, MW 0.58 (0.56–0.60)	< 0.01
Patients receiving norepinephrine (*n*; %)	1249; 82.9% (80.9–84.8%)	1000; 66.4% (64.0–68.8%)	< 0.01, RR 1.25 (1.20–1.30)	< 0.01, OR 1.17 (1.14–1.21)
Patients receiving methylene blue (*n*; %)	147; 9.8% (8.3–11.4%)	153; 10.2% (8.7–11.8%)	> 0.20	> 0.20

This table shows the occurrence rate of the primary endpoint vasoplegia and the total amount of chosen vasopressor drugs between the fine filter and control filter group. Rate of vasoplegia was not significantly different between the fine filter and the control filter cohort

Data are presented as the mean ± standard error, as the median (first quartile–third quartile), as the percentage rates (with 95% confidence intervals), or as the number (*n*) of patients, where indicated

^a^*P* values were calculated using the Wilcoxon-Mann-Whitney *U* test for equality of means, Pearson’s chi-square test, or Fisher’s exact test, as appropriate. Risk ratios or Mann-Whitney effect estimators (Delong method for AUC) are provided as appropriate

^b^*P* values were calculated using the Wald test. The regression model includes cohort, age, sex, and surgery as regression variables. Odds ratios are provided if appropriate and if the *P* value is significant

### Secondary endpoints

The fine filter cohort showed significantly improved lung function by a higher minimal Horowitz index (median 206 vs 191; *P* = 0.04) and lower ARDS score (median 1 vs 2; *P* = 0.01) (Table 3). The maximum creatinine value (median 1.09 vs 1.12; *P* = 0.19) and our modified AKI score rate (11.8% vs 13.7%; *P* = 0.11) showed no significant difference between the two cohorts. No difference was found for delirium score or multi-organ dysfunction scores in the non-parametric analysis but obtained for delirium score (*P* <  0.01) and SAPS II score (*P* <  0.01) in the additional multivariate analysis when taking into account also the baseline values, age, sex, and surgery group (Table 3).

**Table 3:** Secondary outcome parameters (multiple-organ dysfunction, lung dysfunction, acute kidney injury, brain dysfunction)

	Fine filter cohort (*n* = 1506)	Control filter cohort (*n* = 1506)	*P* value^a^	*P* value^b^
Multi-organ dysfunction				
Maximal SOFA score	9 (6–13)	10 (6–13)	> 0.20	0.15
Maximal SAPS II score	53 (39–71)	52 (34–72.25)	> 0.20	< 0.01^c^
Maximal TISS-10 score	23 (18–29.75)	26 (18–30)	0.08	0.06
Maximal TISS-28 score	44 (36.25–51)	45 (36–51)	> 0.20	0.12
Lung dysfunction				
Minimal Horowitz index	206 (119–290)	191 (104.75–280)	0.04, MW 0.52 (0.50–0.55)	0.02
ARDS score	1 (1–2)	2 (1–2)	0.01, MW 0.47 (0.45–0.49)	< 0.01
0, no ARDS (*n*; %)	296; 22.6%	215; 19.6%		
1, mild ARDS (*n*; %)	376; 28.8%	302; 27.6%		
2, moderate ARDS (*n*; %)	377; 28.8%	324; 29.6%		
3, severe ARDS (*n*; %)	258; 19.7%	255; 23.3%		
Heart/lung dysfunction (ECMO)				
Patients on ECMO (*n*; %)	30; 2.0% (1.3–2.8%)	38; 2.5% (1.8–3.4%)	> 0.20	> 0.20
Cumulative duration (days) without first day	4.5 (1.4–7.5)	6.8 (4.8–12.4)	0.01, MW 0.33 (0.19–0.46)	0.09
Cumulative duration (days)	5.5 (2.4–8.5)	8.2 (5.8–13.4)	0.02, MW 0.33 (0.20–0.47)	0.15
Acute kidney injury (AKI)				
Maximal creatinine value before dialysis (mg/dl)	1.09 (0.84–1.74)	1.12 (0.84–1.83)	0.19	< 0.01^d^
Maximal creatinine value before dialysis per interval (*n*; %)	626; 41.9%	600; 40.2%	–	–
0.00–1.00 mg/dl	578; 38.7%	574; 38.5%		
1.01–2.00 mg/dl	169; 11.3%	167; 11.2%		
2.01–3.00 mg/dl	63; 4.2%	79; 5.3%		
3.01–4.00 mg/dl	44; 2.9%	51; 3.4%		
4.01–6.00 mg/dl	13; 0.9%	20; 1.3%		
> 6.00 mg/dl				
Modified AKIN score rate (no AKI/mild AKI vs moderate AKI/severe AKI)	11.8% (10.2–13.5%)	13.7% (12.0–15.5%)	0.11	0.06
Modified AKIN score per classes	1 (1–1)	1 (1–1)	0.11	0.05
0, no AKI (*n*; %)	1; 0.1%	2; 0.1%		
1, mild AKI (*n*; %)	1316; 88.1%	1284; 86.1%		
2, moderate AKI (*n*; %)	92; 6.2%	101; 6.8%		
3, severe AKI (*n*; %)	84; 5.6%	104; 7.0%		
Dialysis rate (*n*; %)	235; 15.6% (13.8–17.5%)	247; 16.4% (14.6–18.4%)	> 0.20	> 0.20
Brain dysfunction				
Maximal delirium score	4 (1–7)	3 (1–6)	> 0.20	< 0.01^e^

This table shows the values of the secondary endpoints multi-organ, heart, lung, and brain dysfunction between the fine filter and control filter cohort. Lung (Horowitz value and ARDS score) dysfunction was significantly better for the fine filter cohort

Data are presented as the median (first quartile–third quartile), as the percentage rates (with 95% confidence intervals), or as the number (*n*) of patients, where indicated

^a^*P* values were calculated using the Wilcoxon-Mann-Whitney *U* test for equality of means, Pearson’s chi-square test, or Fisher’s exact test, as appropriate. Mann-Whitney effect estimators (Delong method for AUC) and risk ratios are provided as appropriate and if the *P* value is significant

^b^*P* values were calculated using the Wald test. The regression model includes baseline, cohort, age, sex, and surgery as regression variables. Odds ratios are provided if appropriate and if the *P* value is significant

^c^Maximal SAPS II score was better for the fine filter cohort

^d^Maximal creatinine value was better for the fine filter cohort

^e^Maximal delirium score was better for the fine filter cohort

The maximum IL-6 value as marker for inflammation was significantly lower in the fine filter cohort (median of 471.5 vs 540.5 ng/l; *P* = 0.01) (Table 4). We hypothesised that specific subgroups of patients with a reduced immune response might especially benefit from the use of in-line filtration with finer 0.2 and 1.2 μm filters. Therefore, we conducted an additional subgroup analysis for cardiac surgery group patients only and all other remaining surgery group patients, regarding their IL-6 values. We obtained the following results in the additional subgroup analysis: *P* = 0.02 and MW = 0.46 (0.43–0.49) for the only cardiac surgery group vs *P* = 0.17 and MW = 0.48 (0.45–0.50) for the remaining patient group. This showed a lower IL-6 value for the finer filter cohort within the cardiac surgery subgroup. In the other subgroup, the difference was not significant, although the tendency of the MW effect estimator shows again a lower Il-6 value for the finer filter. We compared the effect estimators between the only cardiac and the remaining subgroup (with the appropriate chi-square test for AUCs, using the estimator and its standard deviation) and obtained no statistically significant different result (*P* > 0.20). Only a higher number of patients might reveal the estimated significant difference here. In addition, we assessed the IL-6 kinetics by comparing the period including IL-6 maximum value. We obtained as typical peak time a median of 3 (0–12) h for the fine filter cohort and 3 (0–13) h for the control filter cohort (Mann-Whitney test, *P* = 0.19, MW = 0.49 (0.47–0.51)). In this respect, the difference between the two cohorts cannot be explained by different IL-6 kinetics but more likely by the actual maximal value.

**Table 4:** Secondary outcome parameters (inflammation, cytokines)

	Fine filter cohort (*n* = 1506)	Control filter cohort (*n* = 1506)	*P* value^a^	*P* value^b^
Inflammation				
Maximal interleukin-6 value (ng/l)	471.5 (258.8–1062.8)	540.5 (284.5–1147.5)	0.01, MW 0.47 (0.45–0.49)	0.01
Maximal interleukin-6 per interval (*n*; %)			–	–
0.0–50.0 ng/l	30; 2.0%	39; 2.6%		
50.1–200.0 ng/l	188; 15.4%	188; 12.7%		
200.1–500.00 ng/l	519; 35.0%	474; 32.0%		
> 500.00 ng/l	726; 47.6%	778; 52.6%		

This table shows the values of the secondary endpoint inflammation between the fine filter and control filter cohort. Interleukin-6 was significantly better for the fine filter cohort

Data are presented as the median (first quartile–third quartile), as the percentage rates (with 95% confidence intervals), or as the number (*n*) of patients, where indicated

^a^*P* values were calculated using the Wilcoxon-Mann-Whitney *U* test for equality of mean. Mann-Whitney effect estimators (Delong method for AUC) are provided if the *P* value is significant

^b^*P* values were calculated using the Wald test. The regression model includes baseline, cohort, age, sex, and surgery as regression variables. Odds ratios are provided if appropriate and if the *P* value is significant

The length of both cumulated ICU (median 1.2 vs 1.7 days; *P* < 0.01) and hospital stay (median 14.0 vs 14.8; *P* = 0.01) was significantly shorter in the fine filter cohort. Considering the length of stay as a competing risk for death, analysis showed a significantly reduced length of stay in the fine filter cohort also for the survivors (ICU: median 1.0 vs 1.3 days; *P* < 0.01; hospital stay: 14.0 vs 14.4; *P* = 0.01) (Table 5).

**Table 5:** Secondary outcome parameters (length of stay, mortality, in-hospital complications, and morbidity rates)

	Fine filter cohort (*n* = 1506)	Control filter cohort (*n* = 1506)	*P* value^a^	*P* value^b^
Length of stay				
ICU stay (days)	1.2 (0.6–4.9)	1.7 (0.8–6.9)	< 0.01, MW 0.46 (0.44–0.48)	0.02
ICU stay survivors (days)	1.0 (0.6–3.9)	1.3 (0.7–5.2)	< 0.01, MW 0.46 (0.44–0.49)	< 0.01
ICU stay non-survivors (days)	4.9 (2.0–15.3)	7.2 (2.5–17.5)	0.11	> 0.20
In-hospital stay (days)	14.0 (9.2–22.2)	14.8 (10.0–26.8)	0.01, MW 0.47 (0.45–0.49)	> 0.20
In-hospital stay survivors (days)	14.0 (9.2–24.0)	14.4 (10.1–26.2)	0.01, MW 0.46 (0.44–0.49)	0.18
In-hospital stay non-survivors (days)	14.2 (5.6–36.0)	16.0 (8.2–32.4)	> 0.20	> 0.20
Mortality				
In-hospital 28-day mortality rate (*n*; %)	133; 8.8% (7.4–10.4%)	155; 10.3% (8.8–11.9%)	0.19	0.05
In-hospital mortality rate (*n*; %)	198; 13.1% (11.5–15.0%)	213; 14.1% (12.4–16.0%)	> 0.20	0.15
In-hospital complications and morbidities				
Myocardial infarction (*n*; %)	26; 1.7% (1.1–2.5%)	25; 1.7% (1.1–2.4%)	> 0.20	> 0.20
Ischemic stroke (*n*; %)	35; 2.3% (1.6–3.2%)	21; 1.4% (0.9–2.1%)	0.08	0.06
Pneumonia (*n*; %)	172; 11.4% (9.9–13.1%)	217; 14.4% (12.7–16.3%)	0.02, RR 0.79 (0.66–0.96)	< 0.01, OR 0.73 (0.58–0.91)
Sepsis (*n*; %)	145; 9.6% (8.2–11.2%)	183; 12.2% (10.5–13.9%)	0.03, RR 0.79 (0.64–0.97)	0.01, OR 0.73 (0.57–0.94)
Composite endpoint^c^ (*n*; %)	405; 26.9% (24.7–29.2%)	437; 29.0% (26.7–31.4%)	> 0.20	0.05, OR 0.84 (0.71–1.00)

This table shows the values of the co-secondary endpoints length of stay, mortality, and perioperative complications/morbidities between the fine filter and the control filter cohort. Length of ICU and in-hospital stay, pneumonia, and sepsis rate were significantly better for the fine filter cohort

Data are presented as the median (first quartile–third quartile), as the percentage rates (with 95% confidence intervals), or as the number (*n*) of patients, where indicated

^a^*P* values were calculated using the Wilcoxon-Mann-Whitney *U* test for equality of means, Pearson’s chi-square test, or Fisher’s exact test, as appropriate. Mann-Whitney effect estimator (Delong method for AUC) and risk ratios are provided as appropriate and if the *P* value is significant

^b^*P* values were calculated using the Wald test, as appropriate. The regression model includes cohort, age, sex, and surgery as regression variables. Odds ratios are provided if appropriate and if the *P* value is significant

^c^In-hospital mortality, myocardial infarction, ischemic stroke, pneumonia, and sepsis

The overall in-hospital mortality rate was comparable (13.1% vs 14.1%; *P* > 0.20). Similarly, the 28-day mortality was comparable (8.8% vs 10.3%; *P* = 0.19) (Table 5).

Analysing routine ICD-10 codes, typical in-hospital complications were significantly lower in the fine filter cohort (pneumonia 11.4% vs 14.4%, *P* = 0.02, and sepsis 9.6% vs 12.2%, *P* = 0.03) (Table 5). For rarer complications such as ischemic stroke (1.7% vs 1.7%; *P* > 0.20) and myocardial infarction (2.3% vs 1.4%; *P* = 0.06), no significant difference was found (Table 5) between the two cohorts. In addition, a composite endpoint including in-hospital mortality, myocardial infarction, ischemic stroke, pneumonia, and sepsis showed no significant difference between the two cohorts (26.9% vs 29.0%; *P* >  0.20) (Table 5).

## Discussion

In our propensity-matched cohort analysis, the routine use of finer 0.2 and 1.2 μm in-line filters compared to larger 5 μm control filters had no beneficial effects on the rate of catecholamines and on AKI, but was associated with decreased markers of inflammation and a decreased risk of respiratory dysfunction, sepsis, pneumonia, and slightly reduced length of ICU and in-hospital stay. In this respect, we postulate a clinical relevance for patients on intensive care units.

Microparticle-contaminated infusions and drugs may have adverse effects in critically ill patients and are suspected to be responsible for unspecific inflammation that might lead to different organ manifestations. Post-mortem studies have demonstrated that a high amount of particles enters the patient if no in-line filters have been used. This might already be challenging for a healthy patient but could be even worse for severely ill patients. So far, no significant benefits of finer 0.2 and 1.2 μm in-line filters in comparison to no filters were found in a previous adult study [35].

Recent studies including critically ill children and new-borns instead did show a significant reduction of organ dysfunction by using in-line filters (finer 0.2 μm for aqueous solutions and 1.2 μm for lipid-containing mixtures in the filter cohort [9–11] as well as only 0.2 μm for aqueous solutions but no filters for lipid-containing mixtures in the filter cohort [12]). Interestingly, all mentioned studies used no filter in the control cohort compared to the larger 5 μm filter as intervention but that was used in our control cohort.

We initially chose to investigate a possible and clinical important manifestation of inflammation, vascular dilatation, leading to severe vasoplegia. However, vasoplegia is a complex syndrome and we tried to identify this by simply choosing high dose norepinephrine as a surrogate parameter. As a limitation, norepinephrine alone might not be the best surrogate for vasoplegia, as other reasons on a surgical ICU such as ongoing blood loss and other forms of shock might have an additional effect on our readout system.

We explain the positive outcome of some secondary endpoints by the fact that these data are also theoretically able to model manifestations of inflammation. It has to be stated that all our readout systems do not have a proven direct link to microparticle infusion. To the best of our knowledge, the underlying mechanisms are not yet understood. The clinical data at this moment only support the hypothesis that infused particles somehow augment a systemic inflammation response. Therefore, specific subgroups of patients with a reduced immune response might especially benefit from the use of in-line filtration with finer 0.2 and 1.2 μm filters. Our additional subgroup analysis showing significantly better results for IL-6 values for the fine filter cohort within the only cardiac surgery group supports this assumption, comparable to results of Sasse et al. [11]. Exaggerated inflammation levels after cardiopulmonary bypass surgery are a well-known phenomenon, and additional triggers of inflammation should be avoided if possible. Based on our additional basic IL-6 kinetics analysis on all included patients, we conclude furthermore that the beneficial effects of the finer in-line filters are more likely given by reducing the maximal IL-6 value than by reducing the peak time to maximal inflammation.

Based on these data, the benefits for (finer) filters will remain uncertain. We hope that prospective studies will address the theory of microparticle-induced inflammation and evaluate the effects of the finer filters.

### Limitations

Our study had several limitations. As the study was retrospective and depended on an automated readout system, no additional data could be gathered afterwards. Besides that, being a mono-centre study, composition of patients could vary in other centres but we assume, as many results hold also for various subgroups, that this difference is not essential. Furthermore, our analysis includes mainly surgical ICU patients. The accuracy of the encoded routine ICD-10 diagnoses in general has not been assessed, but we assume that for this short time period of 2 years, the coding pattern did not change relevantly. A simple intraoperative factor we cannot statistically exclude and which might be a potential bias could be seen in different surgical and anaesthesiologic teams performing the procedures. Therefore, especially, fluid management regarding type and amount could differ between the time periods. Unfortunately, we cannot normalise the amount of fluids administered to body weight, as we do not have all information to types and volumes given. But we assume due to equal pair matching in each cohort that the received fluids were approximately the same, given the high number of patients which help avoiding bigger random effects. At any time period, mostly crystalloid fluids were used in general. As we did not analyse data regarding urine output, we have to acknowledge that we lose a significant percentage of AKI cases. Furthermore, we do not have additional data about ventilation length and patients’ oxygenation.

Advantages of our propensity-matched study design are mainly statistical: first, the large number of potentially interesting endpoints being investigated and the high number of patients included that did not need to be enrolled, and second, the retrospective approach using routine data which saved time, staff, and costs. Due to the fact that we matched the cohorts equally in number, age, sex, and surgery discipline, at least no bias due to these patient characteristics should have arisen. However, we cannot exclude mild differences regarding other potentially important characteristics such as comorbidities.

## Conclusions

We suggest that in-line filtration with finer 0.2 and 1.2 μm filters could reduce systemic inflammation and maybe morbidity in critically ill adult patients and hence improve safety of intensive care therapy. But further prospective studies might be warranted to investigate these effects in detail.

## Data Availability

The data that support the findings of this study are available from the corresponding author upon reasonable request.

## Abbreviations

AKI: Acute kidney injury
AKIN: Acute Kidney Injury Network
ARDS: Acute respiratory distress syndrome
AUC: Area under the ROC curve
CRF/eCRF: Case report forms/electronical case report forms
ECLIA: Electro-chemiluminescence immunoassay
ECMO: Extracorporeal membrane oxygenation
ICDSC: The Intensive Care Delirium Screening Checklist
ICU: Intensive care unit
KDIGO: Kidney Disease Improving Global Outcomes
MW: Mann-Whitney *U* estimator
OR: Odds ratio
RR: Risk ratio
SAP: Statistical analysis plan
SAPS: Simplified Acute Physiology Score
SE: Standard error
SIRS: Systemic inflammatory response syndrome
SOFA: Sequential Organ Failure Assessment score
TISS: Therapeutic Intervention Scoring System
